# Adeno-associated virus-mediated expression of growth-associated protein-43 aggravates retinal ganglion cell death in experimental chronic glaucomatous injury

**Published:** 2013-06-27

**Authors:** Chukai Huang, Ling-Ping Cen, Lifang Liu, Simone G. Leaver, Alan R. Harvey, Qi Cui, Chi Pui Pang, Mingzhi Zhang

**Affiliations:** 1 Joint Shantou International Eye Center of Shantou University and The Chinese University of Hong Kong, Shantou, P. R. China; 2 Department of Ophthalmology and Visual Sciences, Faculty of Medicine, The Chinese University of Hong Kong, Hong Kong, P. R. China; 3 School of Anatomy, Physiology and Human Biology, The University of Western Australia, Crawley, WA, Australia

## Abstract

**Purpose:**

To examine whether adeno-associated virus (AAV) vector-mediated overexpression of growth-associated protein-43 (GAP-43) has protective or deleterious effects on retinal ganglion cell (RGC) survival in laser-induced chronic intraocular pressure (IOP) elevation injury.

**Methods:**

Adult Fischer 344 rats received unilateral intravitreal injection of either normal saline, AAV-green fluorescent protein (AAV-GFP), or a bi*cis*tronic AAV vector encoding GAP-43 and GFP (AAV-GAP-43). Two weeks later, experimental chronic glaucoma was induced in the injected eyes by scarring the trabecular meshwork with a diode laser. IOP was measured with an impact (rebound) tonometer. Survival of RGCs was estimated after 3 weeks of IOP elevation by quantifying β-III tubulin^+^ neurons in retinal whole mounts. The transfection efficiency of target genes was assessed with direct view of GFP and western blot analysis of GAP-43.

**Results:**

Quantification of β-III tubulin^+^ immunostaining revealed that, compared to uninjured eyes (1,172±80 cells/mm^2^), 3 weeks of laser-induced IOP elevation led to a 60% decline in RGC survival (496±136 cells/mm^2^). Transfection with control vector AAV-GFP by itself did not have a significant effect on RGC viability (468±124 cells/mm^2^). Overexpression of GAP-43 in RGC cell bodies and axons via bi*cis*tronic AAV-GAP-43 led to more severe RGC death (260±112 cells/mm^2^) in IOP elevated eyes, an 80% loss of the total RGC population.

**Conclusions:**

Overexpression of GAP-43 aggravated RGC death in experimental chronic IOP elevation injury. GAP-43 was upregulated in RGCs regenerating after optic nerve injury. Thus, the finding that this same protein is deleterious to RGC viability after chronic IOP elevation may aid in understanding the mechanisms involved in RGC loss in glaucoma and how best to treat this condition.

## Introduction

Glaucoma is the second most common cause of blindness worldwide, and is becoming increasingly prevalent as people live longer [1,2]. The typical excavated appearance of the optic nerve head (ONH) and the loss of retinal ganglion cells (RGCs) attributed to apoptosis [3] are characteristics of pathologic changes in glaucoma. In addition to older age, elevation of intraocular pressure (IOP) is believed to be the major risk factor for glaucomatous RGC loss [4,5], as lowering of IOP has been shown to significantly reduce the rate of glaucoma progression in clinical trials [6], though the mechanism underlying RGC loss has not been clearly elucidated.

It has been proposed that modifications within the axonal microarchitecture and disturbances of axonal transport play important roles in apoptotic RGC death [7,8], caused by direct compression of RGC axons as a result of increased IOP, especially in regions across the lamina cribrosa [9]. Axonal transport is mediated by the axonal cytoskeleton and by motor proteins, including neurofilaments, microtubules, kinesin (anterograde transport), and dynein (retrograde transport) [10,11]. Loss of linear microtubule arrays has been found in compressed axons [12]. A recent study also demonstrated that increased IOP significantly reduces neurofilament heavy (NFH), neurofilament medium (NFM), and neurofilament light (NFL) in the prelaminar, lamina cribrosa, and proximal postlaminar regions compared to a normal eye [7]. Disturbance of the transport system profoundly affects axonal flow, including anterograde transport of mitochondria [13] and retrograde transport of neurotrophins such as brain-derived neurotrophic factor (BDNF) and its receptor trkB [14,15], which are important for RGC viability [16].

Growth-associated protein-43 (GAP-43) is a protein kinase C substrate concentrated in growing axons, growth cones, and synapses in association with actin cytoskeleton [17-19]. It was recently shown that BDNF effects on neuronal survival and plasticity in cortical cultures required the presence of GAP-43, which was related to the F-actin polymerization-depolymerization cycle [20], an important factor in maintaining normal mitochondrial function and preventing cell death [21]. GAP-43 plays an important role in axonal growth. In development or during regeneration after optic nerve injury, GAP-43 is highly expressed in RGCs when they are extending their axons [22,23], and overexpression of GAP-43 in return enhances axonal regeneration in some CNS neurons [24]. There is also evidence that GAP-43 influences neuronal survival [20,25-28]. Importantly, intravitreal injection of BDNF has been reported to increase GAP-43 expression in RGCs threefold [25], which may facilitate neuronal survival [20]. In cortical cells from GAP-43(−/−) animals, where GAP-43 is not present, the neuroprotective effects of BDNF are lost [20]. However, it has been reported that increased expression of GAP-43 can cause the death of adult motoneurons and hippocampal neurons [28,29], and absence of GAP-43 protects sensory neurons from apoptosis induced by trophic factor deprivation [26]. In acute IOP elevation, GAP-43 in RGCs was transiently upregulated up to 72 h after ischemia/reperfusion damage [30], but whether this is relevant to RGC survival is unclear.

We set out to determine if there is a direct effect of increased GAP-43 expression on RGC survival in experimental glaucomatous injury. By using adeno-associated virus (AAV) vectors [31-33], currently the most efficient vector for adult RGC transduction [34], the gene encoding GAP-43 protein was introduced into these retinal cells. The effect of sustained expression of GAP-43 on adult RGC survival was then quantified after laser-induced chronic intraocular pressure (IOP) elevation injury.

## Methods

### Viral vectors

**Figure 1:**
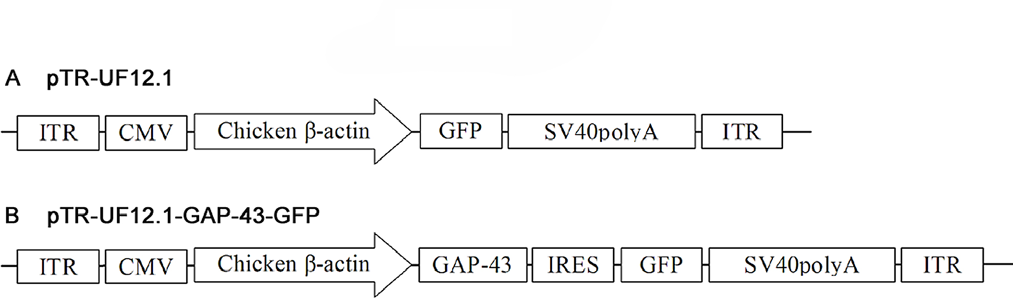
Linear maps of adeno-associated virus (AAV) vector plasmids (based on pTRUF12.1) applied in this study. Transgenes (GFP or GAP-43) were under the control of the cytomegalovirus and chicken β-actin (CMV-CBA) hybrid promoter. GAP-43 plasmids contained IRES that enabled the bi*cis*tronic expression of the GFP reporter gene.

Linear structures of the AAV-2 vector plasmid pTR-UF12.1 used in this study are shown in Figure 1. The expression of GAP-43 was under the control of a hybrid of two promoters: the cytomegalovirus and chicken β-actin (CMV-CBA) [35]. The GAP-43 transgene was linked to a reporter gene, green fluorescent protein (GFP), via an internal ribosome entry site (IRES). In this bi*cis*tronic AAV vector, the GFP gene was therefore expressed after the transcription of the upstream GAP-43 transgene, thus allowing direct identification of transduced RGCs in injected eyes [31-33]. AAV-GFP was used as the vehicle control. The AAV-2 vectors were packaged, and purified according to previously published methodologies [31,36]. Briefly, AAV vector plasmids were co-transfected together with the AAV helper plasmid pDG (provided by J. Kleinschmidt, Heidelberg) into HEK 293T cells (ATCC, Manassas, VA) using a calcium phosphate precipitation method. Viral particles were purified in an iodixanol (Nycomed Pharma, Norway) gradient and finally concentrated on a centricon column (Millipore, MA). Titers (transducing units, TU) were established by transducing 293T cells with a dilution series of the purified AAV and then counting the number of GFP positive cells. The titers of the AAV-GAP-43-GFP and AAV-GFP stocks were in the range of 10^9^ TU/ml. To verify the translation efficiency of transgene GAP-43 encoded in the vector plasmid, HEK 293T cells were transfected directly with the vectors. Twenty-four hours later, cells were fixed with paraformaldehyde and immunostained with antibodies against GFP and GAP-43. GFP was colocalized with GAP-43 protein in the majority of cells.

### Animals

Young adult (8- to 10-week-old) Fischer 344 (F344) rats were used in this study. Rats were housed under standard conditions with a 12 h:12 h light-dark cycle. All experiments conformed to the Association for Research in Vision and Ophthalmology Statement for the Use of Animals in Ophthalmic and Vision Research and were approved by the Hospital Ethics Committee. All possible measures were taken to minimize suffering and limit the number of rats used. All surgery was performed under anesthesia of a 1:1 mixture (1.5 ml/kg) of ketamine (100 mg/ml) and xylazine (20 mg/ml).

### Intraocular injections

Intraocular injection procedures were similar to those described previously [37,38]. Briefly, adult F344 rats were anesthetized by intraperitoneal injection of ketamine and xylazine and topical 1% proparacaine eyedrops. Intraocular injections were made with a posterior approach behind the corneoscleral limbus of the eyeball. A pulled glass needle was connected by polyethylene tubing to a 10 µl Hamilton syringe (Reno, NV) to inject viral vectors or saline. The assembly was prefilled with mineral oil (Sigma Aldrich, St. Louis, MO) before drawing up 5.0 μl of viral vector. The needle was kept in place for several seconds to reduce leakage after withdrawal. Attention was given to avoiding damage to the lens, which is known to cause cataract formation and influence RGC viability [39].

### Laser-induced intraocular elevation

Chronic IOP elevation was achieved by scarring the trabecular meshwork with a 532 nm diode laser. Two weeks after the intravitreal injection of viral vectors or saline, animals were anesthetized as described above. Laser energy was delivered to the trabecular meshwork through a slit lamp on 50–70 spots at 100 µm at a power of 0.7 W and 0.7 s duration. IOP was measured in each eye with the TonoLab (Toilat, Finland), an impact (rebound) tonometer. Using this impact tonometer, IOP was measured in awake and nonsedated animals. Readings were taken before and 1, 3, and 7 days after laser treatment. Measurement of IOP was always performed in the morning between 9:00 and 12:00. Six measurements were taken from each eye and averaged. A second laser treatment was given 1 week later if the IOP difference between the laser and control eye was less than 8 mmHg. IOP was measured weekly for 3 weeks after experimental glaucoma was induced.

### Preparation for histology and examination of green fluorescent protein–expressing cells

The procedures for tissue preparation and immunohistochemistry were also similar to those reported previously [37,38]. The animals were allowed to survive for 3 weeks after laser-induced IOP elevation and 5 weeks after viral vector injection. Then, rats were euthanized with sodium pentobarbitone (150 mg/kg, intraperitoneal injection) and perfused through the heart with cold saline plus heparin followed by 4% paraformaldehyde. Retinas were dissected out from the eyecup and post-fixed in the same fixative for 1 h in a dark chamber. Retinal whole mounts were prepared after post-fixation by transferring the whole retina to a microscope slide. Four relieving incisions were symmetrically made to allow the retinas to flatten. Antifading mounting medium (Dako Corporation, Carpinteria, CA) was used to reduce fluorochrome quenching during analysis by fluorescence microscopy. To determine the total number of viral-transfected cells in each retina, the number of GFP-expressing cells in each field (0.235×0.235 mm^2^) at a fixed distance from one another, in a pattern of grid intersections, was counted. Forty to fifty fields were sampled per retina. The average density of positive cells per field was determined, and the total number obtained by multiplying this figure by the retinal area. Data from different groups were statistically analyzed using the Bonferroni test after one-way analysis of variance (ANOVA) [38,40].

Transfected cells, mostly RGCs and some displaced amacrine cells, were reported mainly located in the ganglion cell layer [31,32]. To identify the latter neurons in the present study, cryosection of the retina and immunohistochemistry using syntaxin antibody that labels amacrine cells was performed. Cryosections of the retinas were cut at 16 µm thickness in nasotemporal orientation after post-fixation for 2 h and cryoprotection in 30% sucrose overnight. The retinal sections were thoroughly washed with PBS (Na_2_HPO_4_·12H_2_O 2.9 g,NaH_2_PO_4_·2H_2_O 0.3 g, NaCl 9.0 g) and blocked with 10% normal goat serum (NGS; Biodesign, Saco, ME) and 0.2% Triton (Sangon Biotech, Shanghai, China) for 1 h. The sections were then immunoreacted with the syntaxin antibody (Dallas, TX; 1:100) overnight at 4 °C. Cy3 (Jackson ImmunoResearch Laboratories, West Grove, PA; 1:400) was used as the secondary antibody for 1 h at room temperature. To identify the location of positive staining in the retina, 4',6-diamidino-2-phenylindole (DAPI; Sigma-Aldrich, St. Louis, MO; 1:2000) was added at the same time as the secondary antibody to stain the nuclei of all cells, thus revealing cellular layers of the retina. Retinal section slices were examined under a fluorescent confocal microscope (Leica TCS SP5-II, Wetzlar, Germany).

### Immunostaining of whole-mount retinas

Immunostaining of whole-mount retinas was essentially as reported previously [40-42]. After the GFP-expressing cells were quantified, the retinas were brushed off the slides followed by three 5-min washes in PBS. Retinas were blocked and permeabilized using 10% goat serum (Biodesign, Saco, ME) and 0.2% Triton X-100 for 1 h. Then the retinas were immunostained overnight at 4 °C with TUJ1 antibody (1:500, anti-β-III tubulin; Covance, Emeryville, CA), which specifically labels adult RGCs in retinal whole mounts [41-44]. To verify the expression of GAP-43 in the retinas, primary antibody for GAP-43 (1:400, Chemicon, Millipore, Billerica, MA) was also applied to the retinas. The retinas were rinsed with PBS (three times for 5 min) and then incubated with conjugated fluorescein isothiocyanate (1:400; Sigma) or cy3 (Jackson ImmunoResearch Laboratories, West Grove, PA; 1:400) secondary antibody overnight at 4 °C. After three washes for 5 min each, the retinas were mounted with antifading mounting medium (Dako, Carpinteria, CA) and examined under the fluorescent microscope. The total number of β-III tubulin^+^ and GAP-43^+^ in each retina was obtained in the same way described above. Data from the different groups were statistically analyzed using the Bonferroni test following one-way ANOVA [38,40].

### Western blots

Western blot experiments were applied to determine the expression levels of GAP-43 5 weeks after viral-vector transfection. After euthanasia with a lethal overdose of anesthesia, optic nerves were quickly dissected out and homogenized using lysis buffer containing 1% NP-40 (Fluka, Nonidet P40 Substitute), 0.25% sodium deoxycholate (Sigma-Aldrich), protease inhibitor cocktail (Roche Applied Science, Mannheim, Germany), Phosphate Stop, and 1 mM phenylmethlsulfonyl fluoride (Sigma, p-7626). Protein concentration was determined using the Bio-Rad (Hercules, CA) protein assay reagent. Approximately 40 µg of protein was loaded and separated in a 10% acrylamide-Bis solution (Bio-Rad) gel. The protein was then transferred onto the nitrocellulose membrane (Amersham Biosciences, Little Chalfont, UK) followed by washing in Tris-Buffered Saline Tween-20 (TBST) and blocking in 5% non-fat milk at room temperature for 1 h. To label the specific protein, the membrane was incubated with anti-GAP-43 antibody (1:400, Chemicon) in 0.5% milk/TBST at 4 °C with shaking overnight. After 2-min washing with TBST five times, horseradish peroxidase–conjugated (HRP) secondary antibody (Covance, Emeryville, CA) was added to the membrane and shaken at room temperature for 1 h, followed by washing in TBST. The labeled proteins were detected using the enhanced chemiluminescence (ECL) reagent (Amersham Biosciences). Light signal from the membranes was detected and captured with the Molecular Imager ChemiDoc XRS System (Bio-Rad). Relative signal densities presenting amounts of target proteins were measured by calculating the band densities with Quantity One 4.6.2 (Bio-Rad), followed by normalization against the housekeeping protein glyceraldehyde 3-phosphate dehydrogenase (GAPDH).

## Results

### Laser-induced intraocular elevation

IOP changes were similar to those seen in previous studies [16,45]. Twenty-five F344 rats were treated with laser unilaterally. The baseline IOP (mean±standard deviation [SD]) before treatment without anesthesia was 11.3±1.9 mmHg and 11.5±1.9 mmHg in the treated and control eyes (n=25), respectively, with the measurement using TonoLab. With the laser treatment, the IOP of all treated eyes increased at least 10 mmHg from baseline IOP. However, the IOP of four eyes returned to normal after 3–4 days. The second laser treatment was applied to these four eyes 1 week after the first treatment. The resultant IOP was again obviously higher than the baseline and remained elevated, but gradually decreased over the longer term. Table 1 shows the average changes in IOP in every group on the treated eye and the control eye for one animal. After laser treatment, the mean IOP of the treated eyes in all three groups was greater than 23 mmHg. However, there was no significant difference in mean IOP among the three treated groups (p>0.05, ANOVA; Table 1). The average peak IOP on the treated eyes of these three groups was higher than 39 mmHg, but no difference was detected between the three groups (p>0.05, ANOVA). In addition, the positive IOP integral difference of the experimental and control eyes among these three groups was not significantly different (p>0.05, ANOVA).

**Table 1:** IOP values in glaucomatous and control eyes.

Treatment Group (n)	Mean IOP (mm Hg)			Peak IOP (mm Hg)		
	Glaucoma	control	Difference	Glaucoma	control	Difference
Laser only (n=8)	28.4±7.4	13.4±1.2	15.1±6.8	45.5±14.8	15.8±1.0	29.8±14.9
AAV-GFP (n=10)	23.7±6.3	11.2±0.9	12.6±6.5	39.9±16.6	13.0±2.3	26.9±16.9
AAV-GAP-43 (n=7)	23.8±4.9	11.4±1.2	12.6±5.3	42.7±8.5	14.3±2.0	29.7±8.8

Data are presented as mean±SD for each treatment group. Mean IOP is the average IOP after induction of experimental glaucoma. Peak IOP is the IOP of the experimental and control eyes on the day that IOP was most elevated after laser treatment.

### Transgene expression in adeno-associated virus–injected eyes

**Figure 2:**
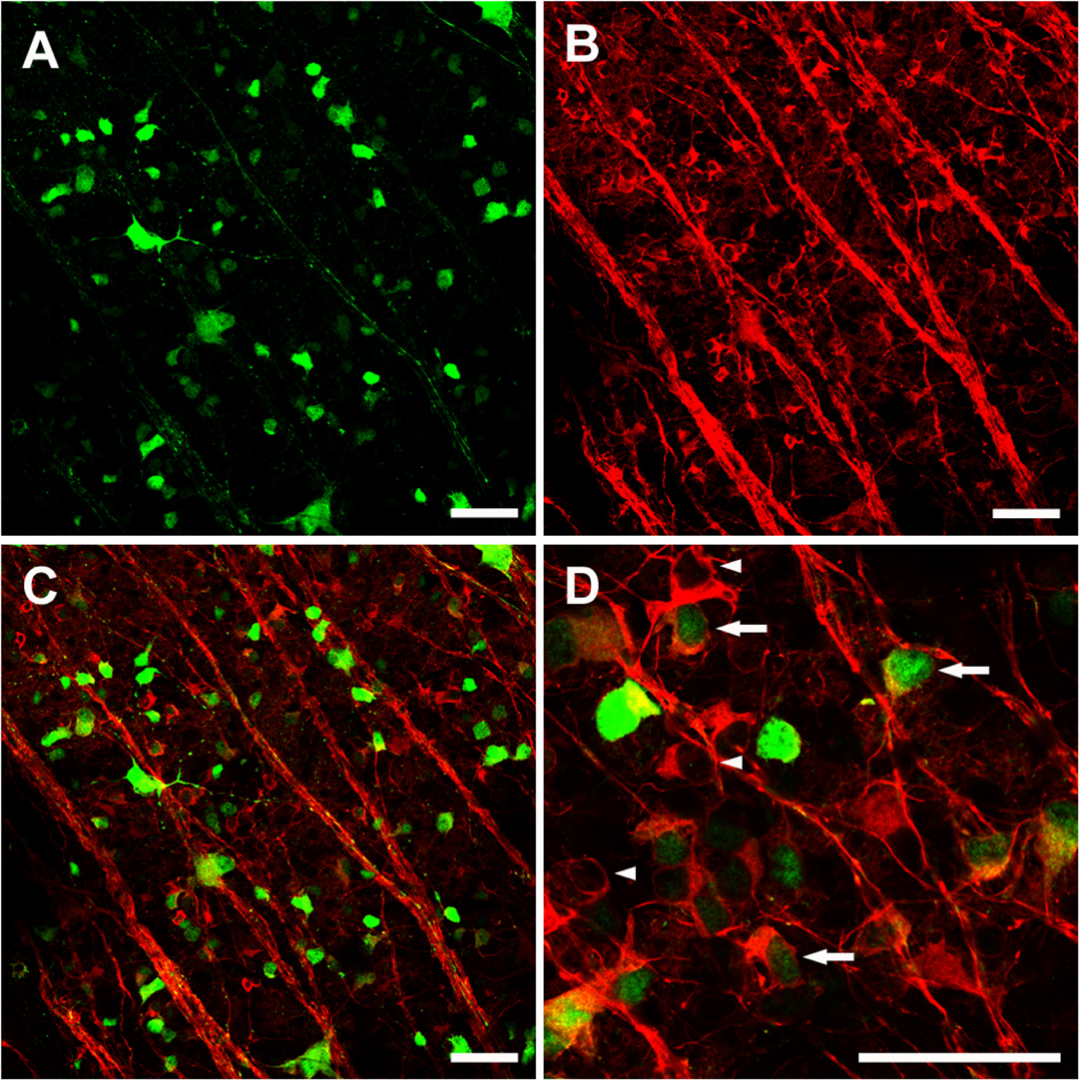
Fluorescent photomicrographs showing characteristics of green fluorescent protein (GFP)^+^ transfected cells and βIII tubulin+ surviving retinal ganglion cells (RGCs) in the same field of retinal whole mount. Five weeks after intravitreous injection of 5.0 μl viral vectors, extensive GFP expression mostly in the RGC layer was shown (**A**). **B** shows βIII tubulin^+^ surviving RGCs in the same field. Merged figure (**C**) of **A** and **B** showing many GFP^+^ cells are also βIII tubulin^+^ and are therefore retinal ganglion cells. **D**: Arrows show examples of transduced RGCs, arrow heads show non-transduced RGCs. Scale bar, 50 μm.

**Figure 3:**
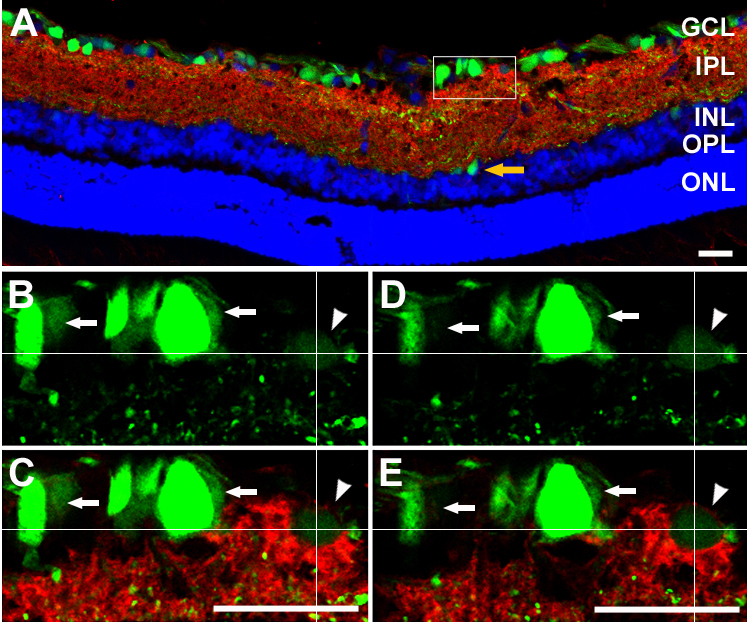
Fluorescent confocal micrographs showing characteristics of green fluorescent protein (GFP)^+^ transfected cells (in green) and syntaxin^+^ cells (in red) in a retinal slice. Most GFP^+^ transfected cells were located in the GCL. Meanwhile, a few transfected cells were also seen in the INL (**A**, yellow arrow). The field in white box (**A**) was scanned at different layers with high resolution confocal microscopy (**B**, **C**, **D**, and **E**). The white thin cross in each figure indicates the same position (Z axis) of scanning. The arrowhead shows the syntaxin^+^ amacrine cell (red) was transfected by the viral vector (green, GFP^+^). White arrows show the GFP^+^ transfected cells without syntaxin staining. Scale bar=25 μm. GCL, ganglion cell layer; IPL, inner plexiform layer; INL, inner nuclear layer; OPL, outer plexiform layer; ONL, outer nuclear layer.

**Figure 4:**
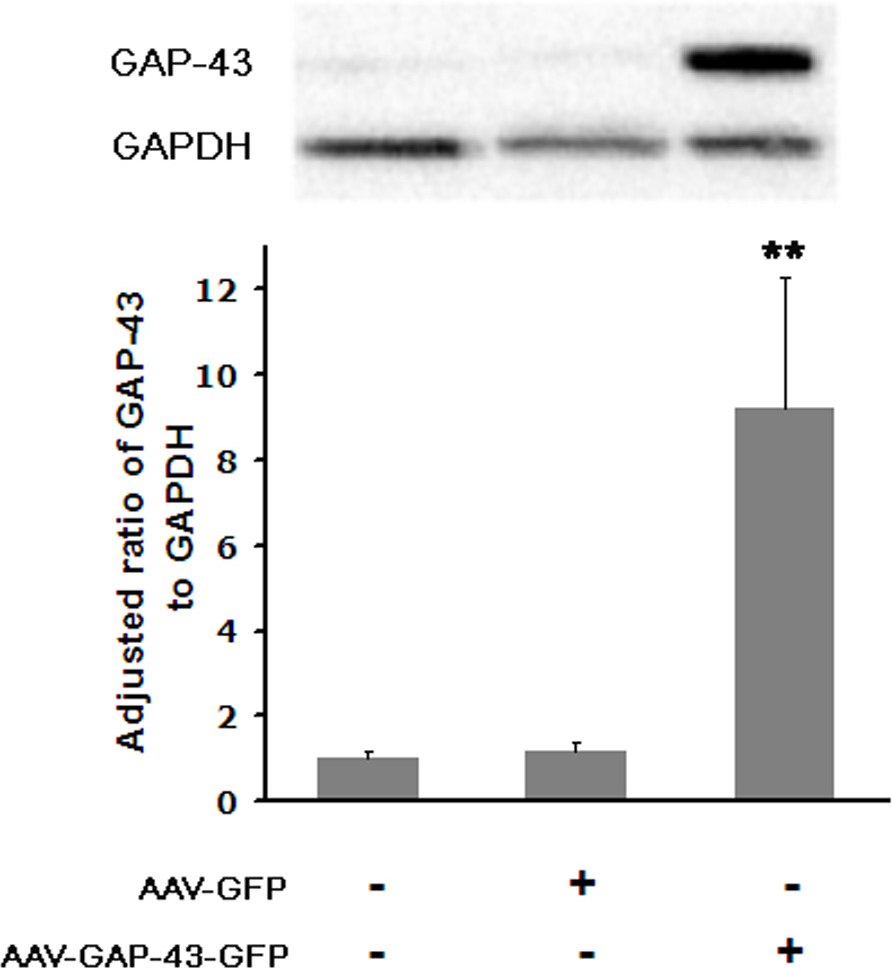
Western blots and analysis of growth-associated protein-43 (GAP-43) expression. Data are presented as an adjusted ratio of GAP-43 to GAPDH. A low level of GAP-43 was expressed in the intact and AAV-GFP groups. With intravitreous injection of AAV-GAP-43, the expression level of GAP-43 in the optic nerve was increased greatly by more than ninefold compared to the AAV-GFP group (**p<0.01, n=3 in each group), error bars=standard deviations (SDs).

To assess the efficiency of transgene expression, bi*cis*tronic AAV-GAP-43-GFP vector and the control AAV-GFP were injected into eyes of non-injured animals containing a normal RGC population; GFP^+^ cells were either directly quantified by examination of retinal whole mounts or observed in retinal cryosection slices with fluorescence confocal microscopy. TUJ1 immunohistochemistry of retinal whole mounts (Figure 2) showed that most of these GFP^+^ cells were RGCs [31,32,46], having the size and dendritic morphology of RGCs and possessing an axon. Fluorescence detection of GFP on retinal sections (Figure 3) revealed AAV-mediated expression of the target gene in cells mostly located in the ganglion cell layer (GCL), while a few were located in the inner nuclear layer (INL, Figure 3A, yellow arrow). Previous studies reported that some displaced amacrine cells could also be transfected in this conditions. In the present study, syntaxin immunostaining detecting amacrine cells was performed (Figure 3) and revealed that some syntaxin^+^ amacrine cells (red, arrowhead) were transfected by the viral vector (green, GFP^+^). Whereas other GFP^+^ transfected cells shown in the field of the white box were syntaxin negative (white arrows). Transfection efficiency was determined only in the identified β-III tubulin^+^ (Figure 2) RGC population. After intravitreal injection of AAV-GFP, about 27% of the β-III tubulin^+^ RGCs were also GFP^+^. The average RGC transfection efficiency was slightly lower in animals with AAV-GAP-43-GFP injection (25%). The transfection efficiency of the target gene was also assessed with western blot analysis of the expression of the GAP-43 protein in the optic nerve, since GAP-43 mainly exerted biologic effects by regulating F-actin polymerization in axons [20]. Blotting and quantitative results for GAP-43 are shown in Figure 4. Data are presented as an adjusted ratio of GAP-43 to GAPDH. As expected, low levels of GAP-43 were expressed in intact animals and rats with transfection of the control viral vector AAV-GFP. After AAV-GAP-43-GFP was intravitreally injected, the expression levels of GAP-43 in optic nerve were dramatically upregulated by more than ninefold compared to the control group (**p<0.01).

### Retinal ganglion cell viability following intraocular elevation

**Figure 5:**
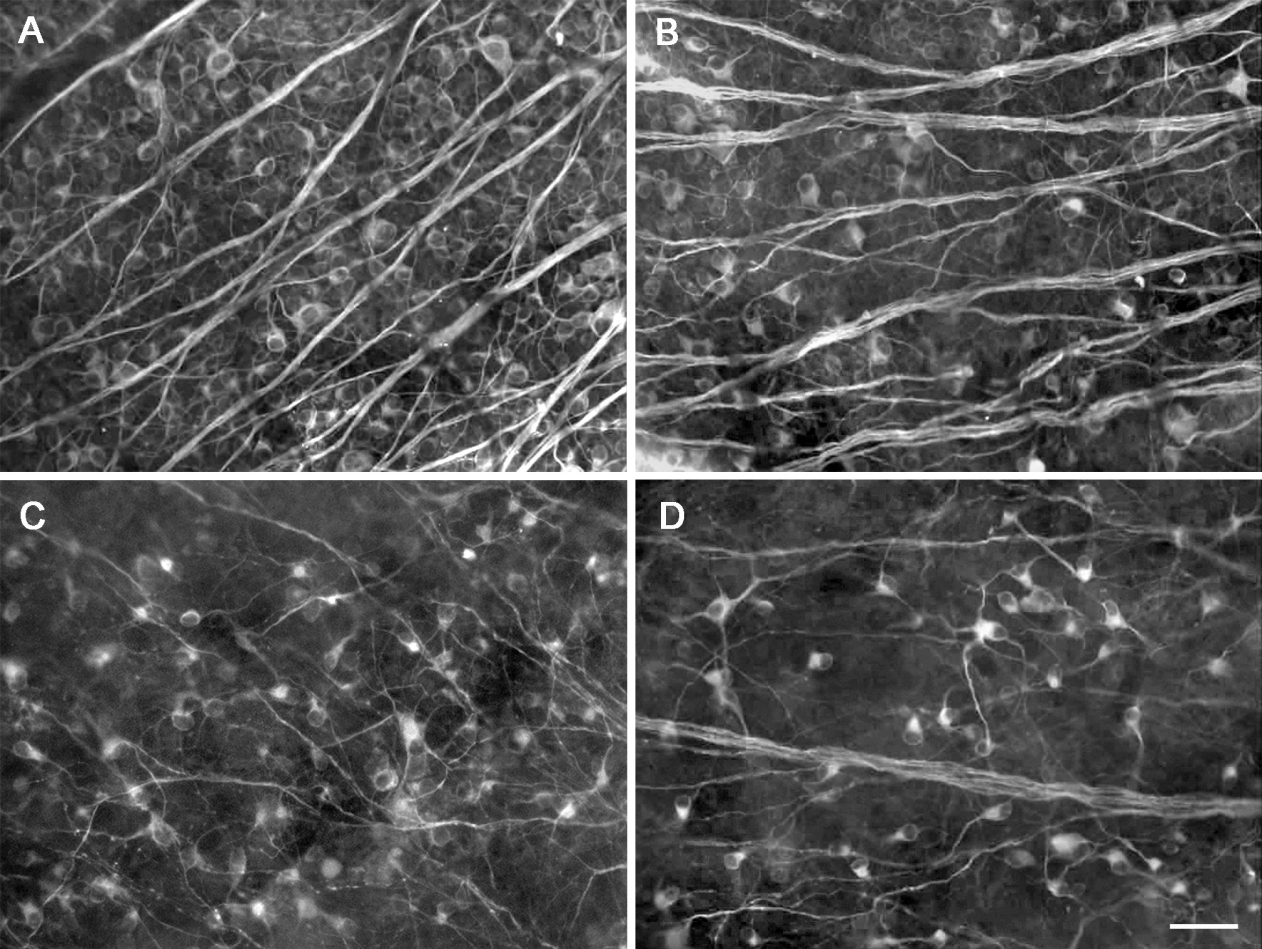
Fluorescent photomicrographs showing characteristics of β-III tubulin^+^ surviving retinal ganglion cells (RGCs) on retinal whole mounts. **A**: Surviving RGCs in the retinas of normal saline group are shown. The retinas in **B**, **C**, and **D** were all treated with laser-induced IOP elevation. Three weeks after laser-induced IOP elevation (**B**), the number of RGCs is significantly reduced compared to the control retinas (**A**). Transfection by control vector AAV-GFP (**C**) did not affect RGC survival, but overexpression of GAP-43 using AAV-GAP-43-GAP (**D**) resulted in a substantial decline in RGC viability. Scale bar=50 μm.

**Figure 6:**
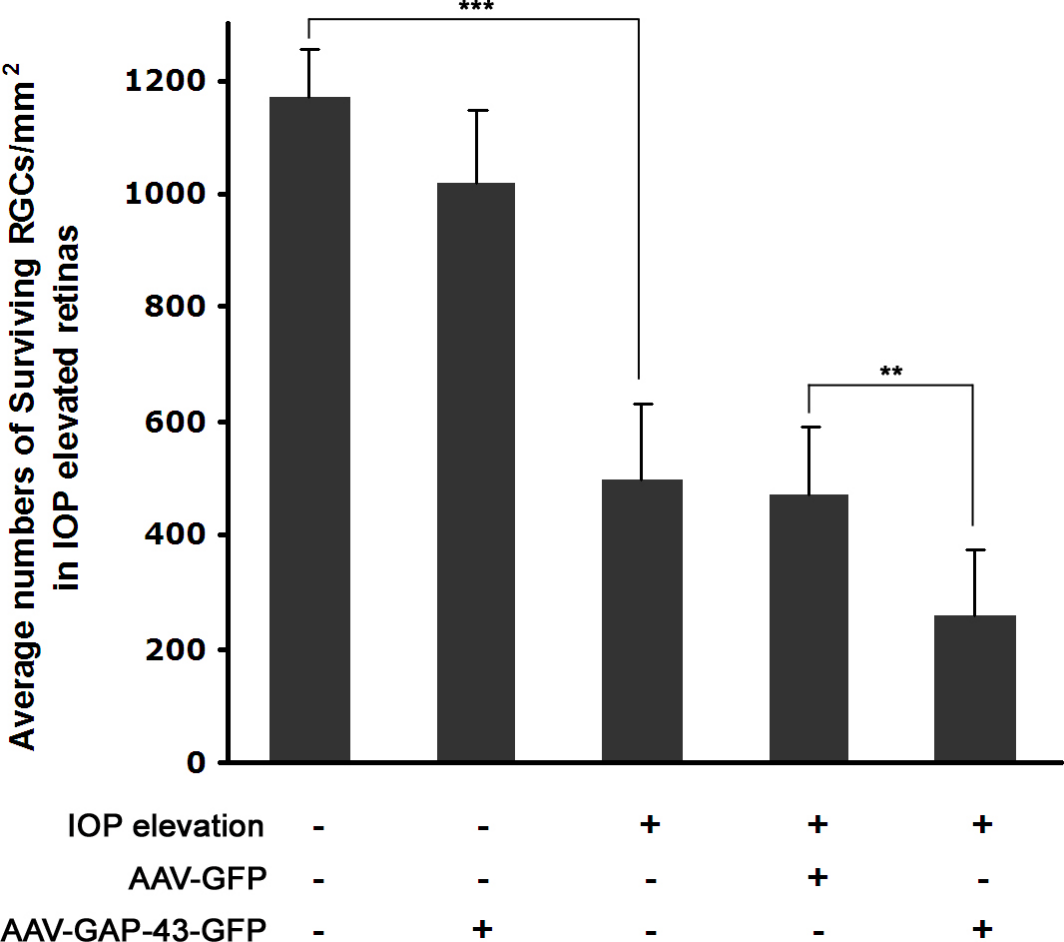
Average numbers of β-III tubulin+ surviving retinal ganglion cells (RGCs) under various experimental conditions 3 weeks after laser-induced IOP elevation. Statistical analysis was conducted against the control groups marked. **p<0.01 and ***p<0.001, Bonferroni test. Error bars=standard deviations (SDs), sample size : n=7, 6, 8, 10, 7 from left to right.

To ensure that the experimental chronic IOP elevation model in the rat was successfully established, the surviving RGC densities in normal saline versus IOP elevation–only groups were quantified. The representative appearance of β-III tubulin^+^ RGCs in retinal whole mounts in intact rats is shown in Figure 5. The average number of RGCs in normal rats was 1172±80/mm^2^ (Figure 5A and Figure 6), which is close to that in a previous study using the same methodology [47]. Quantitative analysis of the surviving β-III tubulin^+^ RGCs was undertaken 3 weeks after laser-induced IOP elevation. A significant (***p<0.001) decline in RGC survival was observed; nearly 60% of the RGCs had died, and only 496±136/mm^2^ (Figure 5B and Figure 6) RGCs survived the chronic injury. Thus, elevated IOP for 3 weeks induced by scarring the trabecular meshwork with laser treatment led to considerable RGC loss and can be used as a chronic glaucomatous model in further investigations.

### Effects of adeno-associated virus-mediated expression of growth-associated protein-43 on retinal ganglion cell survival

Effects of overexpression of GAP-43 on RGC survival in animals with or without laser-induced IOP elevation were examined. In rats without IOP elevation, AAV vectors carrying exogenous gene GAP-43 were injected intravitreally 5 weeks before being euthanized. The average number of surviving RGCs in this group was 1,019±129/mm^2^ (Figure 6), not significantly different from the uninjected, normal group. In animals with induced IOP changes, AAV-GFP (the viral vehicle control) or AAV-GAP-43-GFP was applied to verify the direct effects of overexpression of GAP-43 in RGCs and their axons. Due to the delayed expression of the transgene, caused by the time requirement for converting recombinant AAV-DNA into a transcriptionally active double-stranded form [48,49], viral vectors were injected 2 weeks before IOP elevation. Quantitative analysis of RGC viability is shown in Figure 6. Intravitreal injection of AAV-GFP did not have an obvious effect on RGC survival, and the average number of surviving RGCs (468±124/mm^2^, Figure 5C and Figure 6) was close to that in animals with IOP elevation only. However, in the IOP-elevated eyes, injection of AAV-GAP-43 resulted in a substantial decline in the number of viable RGCs (260±112/mm^2^, **p<0.01; Figure 5D and Figure 6). The minimum number of surviving RGCs counted in individual animals was 140/mm^2^. That is, with overexpression of GAP-43 in cell bodies and axons, RGCs were more vulnerable to laser-induced chronic high IOP injury, leading to 80% RGC loss.

### Comparison of central and peripheral retinal ganglion cell viability

**Figure 7:**
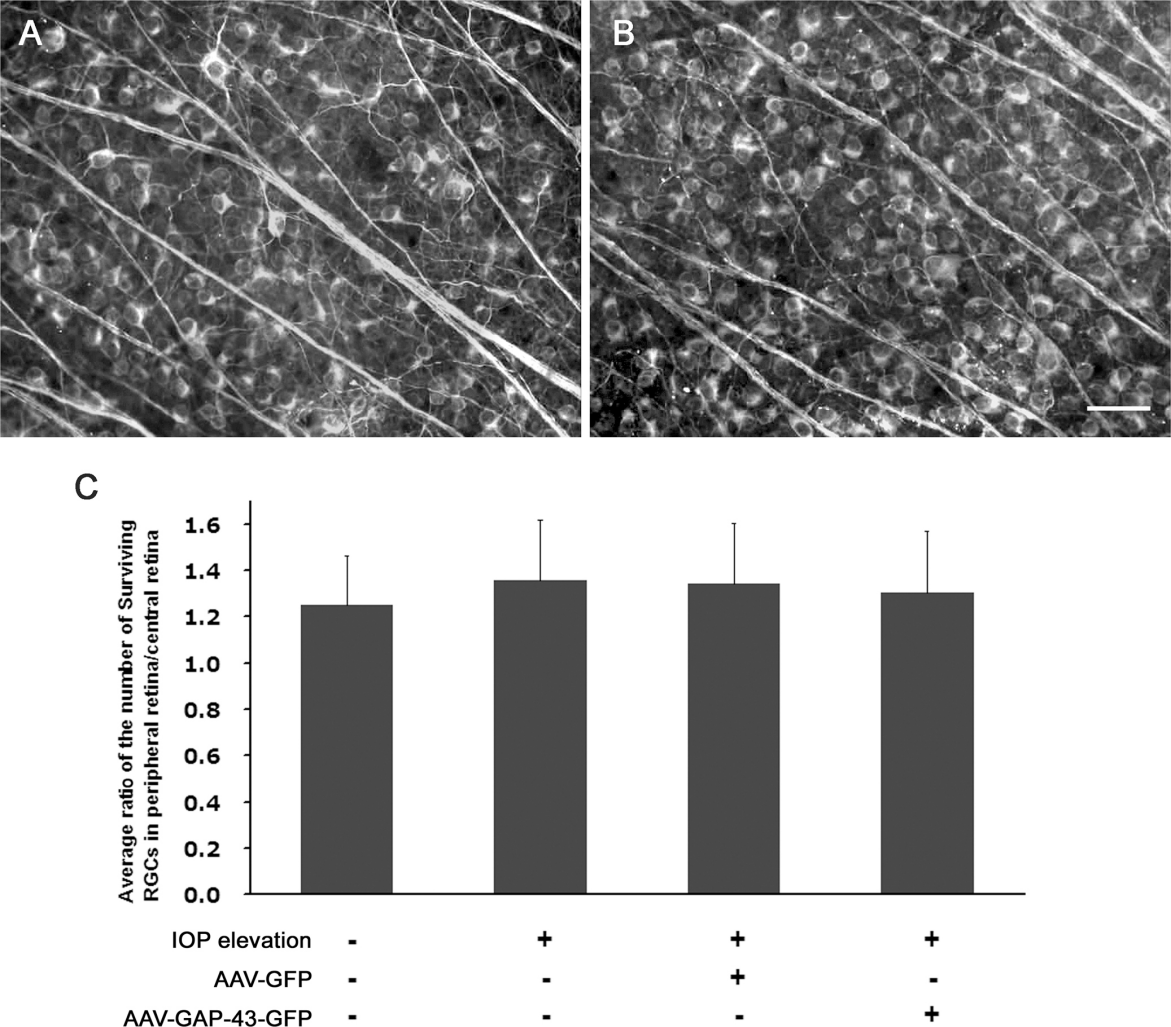
Comparison of central and peripheral retinal ganglion cells (RGCs) viability. Fluorescent photomicrographs of β-III tubulin^+^ surviving RGCs are shown on the central (**A**) and peripheral (**B**) retinas in intact retinas. **C**: Data from various experimental conditions are presented as the ratio of the number of survival RGCs in the peripheral region of the retina to that in central region. Error bars=standard deviations (SDs). Scale bar=50 μm, sample size : n=7, 8, 10, 7 from left to right.

Based on the observations that axonal injury at different distances from the cell body results in different rates of RGC death, specifically, RGCs die largely and rapidly when the ON is transected close to the posterior eye pole, and is delayed as the distance from the transection site to the ON head increases [50,51], we hypothesized that mechanical compression on axons at the ON head induced by IOP elevation may lead to different rates of RGC loss between the central and peripheral retina, and overexpression of GAP-43 in the axon may differentially affect the rates of RGC loss. To examine the differences in RGC viability, regions in the central (0.470 mm from the ON head, Figure 7A) and peripheral (1.175 mm from the ON head, Figure 7B) retina were randomly selected for quantification. Data are presented as ratios of the number of surviving β-III tubulin^+^ RGCs in the peripheral versus central retina. The average ratio in intact retinas was 1.25±0.22 (Figure 7C), showing that the density of RGCs located in the peripheral region was slightly higher than that of the central region. With laser-induced IOP elevation, a somewhat higher average ratio (1.36±0.26) was noticed, but the difference was not statistically significant. In animals with laser-induced IOP elevation, intravitreal injection of AAV-GFP and AAV-GAP-43-GFP did not affect the peripheral/central ratio of RGC viability.

## Discussion

In the present study, we used a gene transfection approach in rats to assess the direct effects of GAP-43 overexpression on adult RGC survival in a laser-induced chronic IOP elevation model. There was a substantial increase in expression of GAP-43 in the optic nerve that appeared to be associated with greater RGC loss in IOP-elevated eyes. These new data indicating GAP-43 as a negative modulator of RGC survival provide a new perspective for interpreting the thus far unexplained loss of neurons overexpressing GAP-43 [26,28,29].

After laser-induced IOP elevation, GAP-43 expression was significantly upregulated but lasted no longer than 1 week [30]. With viral transfection, however, high-level expression of GAP-43 was detected more than 5 weeks later. Cytotoxic effects are obviously a concern when GAP-43 is upregulated much higher than that in normal or in glaucomatous eyes. However, previous work has showed that AAV-mediated upregulation of GAP-43 has no obvious negative effects on RGC viability in studies involving optic nerve crush or transection plus peripheral nerve grafting [31], by comparing surviving RGCs in the transduced area and the non-transduced area of the retina, although increased dendritic complexity was noted [33,52]. In the stimulated regenerative stage after optic nerve transection, the GAP-43-expressing level in the optic nerve was found tremendously upregulated and helped to support axonal regeneration, but no deleterious effects on RGCs were noted [37]. High IOP may trigger some unspecified detrimental mechanisms that are sensitive to increased levels of GAP-43 but are not involved in conditions without IOP elevation. Gagliardini et al. [26] proposed that GAP-43 may be a mediator of semaphorin III–induced growth cone collapse and neuron death. Interestingly then, most class III semaphorins are expressed by RGCs [53], and changes in the expression of some class III semaphorins and their coreceptors have been noted in the retina after optic nerve injury [54]. However, whether this proposed mechanism is relevant to the present retinal injury model remains to be elucidated. Interestingly, in IOP-elevated conditions, some RGCs can survive GAP-43 overexpression for a long period. One explanation is that different types of RGCs may respond differently to the same interventions: The RGCs in a retina are not all the same but have different morphologies and distinct physiologic features that had been described by many RGC classification studies [55-57].

After AAV-GAP-43-GFP injection, only about 25% of the RGCs expressed the target protein; thus, RGC loss would not have been so extensive if overexpression of GAP-43 affected only those RGCs transfected with the vector. GAP-43 has been reported to play crucial roles in regulating synaptic plasticity [29,58], which was also important for maintaining neuronal normal functioning and viability. However, whether overexpressed GAP-43 influences synapse plasticity and therefore leads to more neural cell death is not known. There is evidence that AAV-mediated GAP-43 expression in retinal cells can have measurable effects on untransfected RGCs, subtly changing their dendritic architecture [33,52]. One possibility is that GAP-43 can influence extracellular protease activity by influencing nexin-1 [59], but further studies are needed to clarify, especially in experimental glaucomatous conditions, how GAP-43 overexpression can lead to the loss of non-transfected RGCs.

According to the observation that optic nerve injury closer to the ONH resulted in more rapid and severe RGC loss [50,51], RGCs in the central region of the retina were thought to be more vulnerable than those in the peripheral retina. In the present study, we found that the ratio of surviving RGCs in the central retina to those in the peripheral retina in eyes with high IOP was slightly higher than that in the control eyes, yet the difference was not statistically significant. Unlike complete axotomy in optic nerve transection, with chronic compression at the ONH by elevated IOP [7,8], the injury site on axons at different distances away from neuron cell bodies appears not to be a decisive factor for RGC viability. In addition to the compression at the ONH, high IOP can influence the neuron cell bodies within the retina, which may directly lead to RGC death regardless of the axonal distance to the ONH.

In conclusion, we demonstrated that AAV mediated overexpression of the axonal growth–associated protein GAP-43 in RGCs and severely aggravated RGC death in experimental glaucomatous injury. Thus, in addition to known positive biologic actions on promoting axonal growth, GAP-43 also exerts negative effects on RGC survival in high IOP conditions. This finding thus adds an important aspect to our existing understanding of RGC loss in glaucoma. Specific mechanisms involved thus far have not been well explained. More detailed investigations need to be conducted to obtain full understanding of the molecular pathological processes of glaucoma and therefore possibly provide better treatments.
